# Subjective vision assessment in companion dogs using dogVLQ demonstrates age-associated visual dysfunction

**DOI:** 10.3389/fvets.2023.1244518

**Published:** 2023-08-17

**Authors:** Callie M. Rogers, Michele M. Salzman, Zhanhai Li, Natascha Merten, Leah J. Russell, Hannah K. Lillesand, Freya M. Mowat

**Affiliations:** ^1^Department of Surgical Sciences, School of Veterinary Medicine, University of Wisconsin–Madison, Madison, WI, United States; ^2^Department of Biostatistics and Medical Informatics, University of Wisconsin–Madison, Madison, WI, United States; ^3^Department of Population Health Sciences, School of Medicine and Public Health, University of Wisconsin–Madison, Madison, WI, United States; ^4^Department of Medicine (Geriatrics and Gerontology), School of Medicine and Public Health, University of Wisconsin–Madison, Madison, WI, United States; ^5^Department of Ophthalmology and Visual Sciences, School of Medicine and Public Health, University of Wisconsin–Madison, Madison, WI, United States

**Keywords:** aging, proxy questionnaire, canine, retina, companion dog, low luminance questionnaire, visual function questionnaire

## Abstract

**Introduction:**

Dim light vision as assessed by proxy and clinical tools is commonly impaired in older humans and impacts quality of life. Although proxy visual assessment tools have been developed for dogs, it is unclear if they are sensitive enough to detect subtle visual dysfunction in older dogs. We sought to determine if a newly designed proxy visual function questionnaire could detect age-associated differences in visual behaviors in varying lighting conditions in dogs.

**Methods:**

A 27-item questionnaire (the dog variable lighting questionnaire, dogVLQ) was designed to assess visual behavior in dogs in different lighting settings. We conducted the dogVLQ, a previously validated visual function questionnaire the dog vision impairment score and performed light- and dark-adapted electroretinography (ERG) on a subset of dogs. Questionnaire scores were analyzed for dog age associations using correlation analysis.

**Results:**

Questionnaire responses from 235 dog owners were obtained (122 female, 112 male dogs), 79 of which underwent ERG (43 female, 36 male dogs). Bright light visual behavior was significantly associated with light-adapted bright flash ERG amplitudes, visual behavior in near darkness was associated with dark-adapted ERG amplitudes. The dogVLQ identified worse vision in older dogs in bright light, dim light, and darkness; predicted onset was younger for vision in near darkness. Older dogs had more difficulty navigating transitions between lighting conditions.

**Discussion:**

Subjective dog owner assessment of visual function associates with objective measurement of retinal function in dogs and supports reduced vision-mediated behaviors in older dogs.

## 1. Introduction

Studies in ophthalmology have shown the benefit of standardized recording of patient reported outcome measures, which can inform the impact of disease and the efficacy of interventions (1–3). Particularly in ophthalmology clinical trials or in diseases with a protracted clinical course, the detection of subtle changes to vision and vision-related quality of life (VRQOL) is critical to sensitive outcome assessment.

There are multiple well-validated visual function questionnaires (VFQ) and VRQOL scoring systems that are used for people, with the most common being the National Eye Institute Visual Function Questionnaire (NEI VFQ) (4, 5). This questionnaire can robustly determine the severity of numerous chronic eye conditions including retinal vascular disorders, age-related macular degeneration, and other causes of low vision (6–8). In aging, clinically significant decline in dim-light vision onsets earlier than bright-light vision is compromised (9). Increasing emphasis has therefore been placed on measuring the low luminance visual abilities in older humans (10). This sparked the inception of low luminance questionnaires (LLQ), with subscales related to low luminance settings (11, 12). LLQ scores are significantly associated with clinical measures of visual function in low light settings, such as contrast sensitivity, visual acuity, reading speed and microperimetry, providing a subjective method to assess the impact of age- and disease-related visual impairment on routine activities (12–14). Questionnaire-based assessment is complicated by an inability to effectively communicate, as seen in pre-verbal or non-verbal children and in severely cognitively impaired populations. In these cases, proxy assessment in the form of proxy-reporting questionnaires by parents or caregivers can be implemented (15–20). A children’s visual function questionnaire (15), and a similar questionnaire, the PedsQL 4.0 (16) have been specifically developed with proxy-reporting in mind and depend on parental abilities to subjectively assess their child’s vision and quality of life. Neither the NEI VFQ nor the LLQ are commonly used in groups where proxy-reporting is required.

Veterinary medicine relies on proxy reporting of clinical signs of disease or dysfunction by pet owners. This information allows the measurement of changes in companion animal health and can be applied to research or used for clinical decision making. To date, two proxy visual function questionnaires have been developed for dogs. The first questionnaire, the canine visual function instrument (CVFI), was designed with the purposes of simplicity of completion, and for sensitivity to differentiate dogs with normal vision from those with heritable forms of blindness (21). The CVFI was found to be both reliable and valid in seeking self-reported observations from owners and had high empirical validity in relation to the visual quality scale. A second study developed the “visual impairment score” (VIS); questions were based on human VFQs and common questions asked during clinical visits by veterinary ophthalmologists (22). Internal validity was demonstrated as the instrument could effectively differentiate sighted, unilaterally blind, and bilaterally blind dogs. While capable of detecting extreme vision loss, neither instrument described sensitivity to subtle vision changes, nor validation using objective tests (such as electroretinography, maze navigation skill) or had subsections regarding vision specifically in different lighting environments. Chronic ophthalmic conditions with long clinical courses such as hereditary and age-associated vision loss (23, 24), cataracts (25), and corneal opacification (26) occur in dogs, like in humans. Because the subtle pre-clinical signs are difficult for owners to determine, many dogs present for clinical evaluation late in disease, rendering treatment more challenging. In addition, the effect of aging on dog visually mediated behavior is unknown. A low-luminance questionnaire would be preferred when studying aging, because of the understanding from human aging studies that low luminance visual deficit onsets prior to vision deficit in bright lighting (9). Considering the contribution of vision to cognitive functioning in people, whereby worse vision is associated with worse cognitive function, and predicts more significant future cognitive decline (27–30), the effect of visual decline on dog cognitive assessment will be important to determine in the future.

In this study we aimed to evaluate a novel proxy visual function questionnaire (the dog variable lighting questionnaire; dogVLQ) for assessing vision in high and low lighting situations in dogs. We also wanted to determine if there was variance in questionnaire scores across age groups and if the dogVLQ could be validated using electroretinography. The purpose of our study was to determine the effect of dog age on proxy assessment of visual function in various light settings including bright light, dim light, near darkness, and during transitions between different lighting conditions.

## 2. Methods

### 2.1. Institutional approval

Use of the questionnaire-based proxy assessment was exempted by the Institutional Review Board of the University of Wisconsin-Madison and the UW-Madison Institutional Animal Care and Use Committee. Validation in the subset of dogs in which electroretinography was performed was approved by the Institutional Animal Care and Use Committee (approval number V006521). All dog owners signed an informed consent form prior to enrollment.

### 2.2. Study participants

Questionnaires were disseminated by mail or digitally to pet owners who were invited to participate in a longitudinal study of dog aging based in Wisconsin. Participants were solicited *via* direct email request to known prior participants in clinical research studies with the university, and *via* fliers sent to local veterinary practices. Current dog owners were eligible to participate. Questionnaire participation had no exclusion criteria, provided the contacted owners had a dog living with them. If multiple dogs lived in a household, the dog owner was asked to select the oldest, healthiest dog to describe. Dog owners completed the questionnaire on their own in order to consider and observe their dog’s behavior. A subset of dogs were invited to participate in a clinical visit at the University of Wisconsin-Madison School of Veterinary Medicine, for participation in a study on aging of the visual system and retinal electrophysiologic responses (24). Inclusion criteria for invitation to the clinical visit were dog age over 1 year and health/temperament suited to study procedures without the need for administration of anxiolytic medication (trazadone, gabapentin) which impacts retinal electrophysiologic responses (24, 31).

### 2.3. dogVLQ questionnaire development and content validation

Questions for the dogVLQ were adapted from the human LLQ or were generated to mimic those typically asked in history taking from dog owners for clinical assessment of visual dysfunction in dogs. Consultation with current dog owners and veterinary ophthalmologists was used to incorporate common dog activities and behaviors relevant to different lighting conditions into questionnaire content.

Each dogVLQ question had response options ranging from inability to perform the behavior because of vision (scored as 0), to performs the behavior without any issue relating to vision (scored as 100). There was a total of 5 visual response options (scored 0–100), in addition to two unscored options that allowed respondents to acknowledge that their dog either did not normally perform that behavior or were unable to perform it for reasons other than vision. The dogVLQ was separated into four subsections exploring the dog’s ability to perform activities and interact with their owners in different light settings: bright light, dim light, darkness, and circumstances where dogs transition from one lighting condition to another: from bright to dim light and from dim to bright light.

Preliminary candidate dogVLQ questions (*n* = 25) were shared with evaluators (15 dog owners and 15 veterinarians). Evaluators were asked to rate the relevance of each question and to provide comments regarding clarity of the question, and reasoning for assessment of relevance. Each question was rated on a five-point Likert scale (strongly favorable, favorable, neutral, unfavorable, and strong unfavorable) in specifically assessing vision. Scores were evaluated for clarity and relevance and dichotomized as favorable (strongly favorable, favorable) or unfavorable (unfavorable, strongly unfavorable). Questions that were rated as neutral were not included in either group. A content validation index (CVI) was determined for each question as previously described (22). Questions were excluded if the CVI was ≤0.60. Only favorable questions with no evaluator question of clarity were used in the final questionnaire. Based on the CVI and reviewer feedback, some questions were removed, and some were added (see results section). The final utilized 27-item questionnaire and answer options are provided in Supplementary Information.

dogVLQ section responses were expressed as a numerical score from 0 to 100 and grouped according to section (bright light, dim light, near darkness, and transition between different light intensities). Higher scores therefore denoted better vision. Occasionally, some owners did not provide an answer to all questions or responded that their dog did not perform a specific behavior for reasons unrelated to vision. Scores were therefore adjusted to account for incomplete responses or non-relevant behaviors by dividing the aggregate score for each dogVLQ subsection by the number of questions answered.

### 2.4. Other questionnaire content

The questionnaire contained questions regarding dog date of birth, sex and neutering status. Owners were also asked to complete the previously validated visual impairment score questionnaire. Visual Impairment Score (VIS) responses were scored from 0 to 4 based on published scoring criteria (22). For the VIS, lower scores denote better vision. Occasionally, some owners did not provide an answer to all questions or responded that their dog did not perform a specific behavior for reasons unrelated to vision. Scores were therefore adjusted to account for incomplete responses or non-relevant behaviors by dividing the aggregate score for the VIS by the number of questions answered. To widen the scale of the VIS scores due to this adjustment method, generated VIS scores were multiplied by 100 to create the final adjusted scores.

### 2.5. Clinical examination

All dogs that completed a clinical visit underwent ophthalmic examination by a board-certified veterinary ophthalmologist (FM) including slit lamp biomicroscopy (model SL17: Kowa, Tokyo, Japan), indirect ophthalmoscopy (Welch Allyn, Skaneateles Falls, NY, USA) and tonometry (Icare Tonovet, Icare, Finland). Animals were excluded from ERG evaluation if both eyes had baseline intraocular pressure > 25 mmHg, because of the need for mydriasis for ERG and potential for a mydriasis-associated increase in intraocular pressure (32, 33). The eye for electroretinography was selected based on the clearest ocular media. When both eyes were equally clear, the eye for electroretinography was randomly selected using an open-source online randomizer^1^.

Unilateral electroretinography (ERG) was performed with mydriasis (Tropicamide, 1%, Akorn, Lake Forest, IL; administered at least 40 min prior to initiation of ERG) and topical corneal analgesia (Proparacaine 0.5%, Akorn, Lake Forest, IL), using a handheld ERG unit (RetEval, LKC Technologies, Gaithersburg, MD) as previously described (24), apart from studying a wider light stimulus range. The dogs were first tested in ambient room light with a background light of 30 cd/m^2^, with stimuli consisting of flashes of increasing stimulus strength (light-adapted stimuli at 1, 3, and 10 cds/m^2^; interstimulus interval 0.5 s, 20 flashes averaged), followed by 20 min of dark adaptation and a dark-adapted flash stimulus series (a series of 8 different dark-adapted flash stimuli ranging from 0.003–10 cds/m^2^; interstimulus interval 2 s, 10 averages for 0.003–0.03 cds/m^2^, interstimulus interval 5 s, 8 averages for 0.1–1 cds/m^2^, interstimulus interval 10 s for 3 cds/m^2^, 20 s for 10 cds/m^2^, both 3 averages).

ERG data were grouped based on light-adapted (LA) and dark-adapted (DA) responses. The amplitudes of b-waves of all ERG tracings obtained were measured and compared. The b-wave amplitude was measured from the baseline, or the depth of the a-wave trough (where present) to the peak of the b-wave. The b-wave was selected as it is detectable at a wider range of stimulus strengths to compare to dogVLQ subsection scores (24). The b-wave represents a summed response of the retinal bipolar cells to input from the photoreceptors and represents a measure of both outer and inner retinal function (34).

### 2.6. Statistical analysis

All purebred dogs and dogs that underwent clinical evaluation were assigned an estimated life stage as previously described (24). This was performed to account for the variance in lifespan due to breed. Life stage was assigned using the AAHA dog life stage criteria (35). Dogs were classified as juvenile (<18 months; lifestage 1), young (adult, but <50% of predicted lifespan; lifestage 2), mature (between 50 and 75% of predicted lifespan; lifestage 3), senior (within the last 25% of predicted lifespan; lifestage 4) or geriatric (>100% of predicted lifespan; lifestage 5). Where expected lifespan was unclear in mixed breed dogs, a purebred dog breed of similar size and stature was used to estimate life stage. The distributions of dogVLQ, VIS, ERG, and age were examined by Kolmogorov–Smirnov Tests for normality. This determined that dogVLQ and VIS scores were not normally distributed, while ERG and age were normally distributed. Thus, the correlations between age and dogVLQ/VIS scores and dogVLQ and ERG were examined by Spearman rank correlation, where the magnitude of correlation coefficient showed the strength, and the sign of correlation coefficient showed the direction. The X-intercepts and slopes were also provided if the data were fit by linear regressions. Regression line slope and *Y* = 100 x-intercepts for the dogVLQ and *y* = 0 x-intercept for the VIS were calculated based on the simple linear regression formulae. Only univariate analyses were performed and *p*-values were considered as significant if <0.05. All analyses were done by Statistical Analysis System (SAS) version 9.4, Cary, NC.

## 3. Results

### 3.1. Content validation

An original 25 potential questions were initially assessed for relevance by 11 non-veterinarian dog owners and 10 dog-owning veterinarians, of which 7/10 were veterinary ophthalmologists. One question did not meet content validity criteria (content validation index; CVI 0.52); this question and a related question (asking about an identical behavior in very low lighting, CVI 0.67) were excluded (questions are provided in Supplementary Information). The remainder of the questions had a median CVI of 0.76 (interquartile range 0.71–0.9). A further 4 questions that were proposed to assess vision in darkness or near darkness were excluded (median CVI 0.69), based on consistent feedback from experts that dogs would be unlikely to perform that behavior or hard to assess performing the specific task in that lighting condition (questions are provided in Supplementary Information). For the final distributed questionnaire, the language of 10 questions (5 questions in bright lighting and dim lighting respectively) was modified based on feedback to improve readability and interpretation by the participant. One question (for bright light) was added to the dim light section to make the dim and bright light questions identical bar lighting conditions (question 14). A further 7 questions were added, relating to performing a “high-five” or giving a paw (in bright and dim lighting, questions 7 and 18), catching an object when thrown (in bright and dim lighting, questions 5 and 16), looking at a hand with a treat in it (in bright and dim lighting, questions 6 and 17), and chasing a flashlight in very low lighting (question 24). These new questions did not undergo additional content validation.

### 3.2. Demographics

Demographic information for the questionnaire cohort and the ERG validation subcohort are described in Table 1. A total of *n* = 235 dog owners submitted a completed questionnaire, and *n* = 79 dogs underwent ERG validation. Dogs across the lifespan were recruited (median age 94 months, median lifestage 3, mature adult). The majority of dogs were graded by their owners as medium sized (20–50 lb) or large sized (50–100 lb). Similar numbers of female (52%) and male (48%) dogs were enrolled, with the majority spayed or neutered. Similar numbers of purebred (49%) and mixed breed (51%) dogs were enrolled. Purebred dogs were derived from 44 different purebred breeds (Table 1).

**Table 1:** Demographics of all dogs and the ERG subset.

	All (*n* = 235)	ERG validation subgroup (*n* = 79)
Age, months; median (interquartile range), *n* = 235	94 (60–133)	94 (61–134)
Lifestage, median (interquartile range), *n* = 148	3 (2–4)	3 (2–5)
Weight category (*n*)	Miniature/toy (26) Small (24) Medium (74) Large (109) Giant (2)	Miniature/toy (2) Small (8) Medium (23) Large (46) Giant (0)
Female, *N* (%)	122/234 (52%)	43/79 (54%)
*Spayed, N*	111/121	42/43
*Intact, N*	10/121	1/43
Male, *N* (%)	112/234 (48%)	36/79 (46%)
*Neutered, N*	107/111	34/36
*Intact, N*	4/111	2/36
Purebred, *N* (%)	111/228 (49%) Golden Retriever (20) Labrador Retriever (16) German Shepherd (6) Other breeds (41 different breeds, *n* = 67) Not described (2)	41/79 (52%) Golden Retriever (8) Labrador Retriever (5) German Shepherd (4) Other breeds (18 different breeds, *n* = 24)
Mixed breed, *N* (%)	117/228 (51%)	38/79 (48%)
Median dogVLQ score, *n* = 235	Bright: 100 (97.2–100) Dim: 100 (96.9–100) Near Dark: 100 (87.5–100) BL to DL: 100 (100–100) DL to BL: 100 (100–100)	Bright: 100 (96.4–100) Dim: 100 (96.4–100) Near Dark: 100 (90–100) BL to DL: 100 (100–100) DL to BL: 100 (100–100)
Median VIS score, *n* = 235	12 (0–29)	12 (6–29)

### 3.3. Clinical examination

No animals were excluded for ERG completion because of clinical examination findings. All eyes had intact menace response indicating at least some visual response. No animal or eye had overt glaucoma or intraocular inflammation. Median pre-dilation intraocular pressure for eyes that underwent ERG was 19 mmHg (interquartile range 17–21 mmHg). Clinical examination findings in the cornea of the eyes that underwent ERG included mild stippling or erosion of the epithelial surface (2), focal paraxial or peripheral corneal edema (2), focal stromal opacity (2), focal peripheral endothelial pigment (1) and focal epithelial peripheral pigment (1). Clinical examination findings in the lens included mesenchymal pigment remnants on the anterior lens surface (6), nuclear sclerosis (61), nuclear fibrillar change (16), incipient cortical cataract (15). One dog had bilateral late immature cataracts, but intraocular pressure was not elevated (10 mmHg in both eyes) and an ERG was performed. Clinical examination findings in the vitreous of eyes that underwent ERG included pigmented cells in the anterior vitreous (1), asteroid hyalosis (1) and syneresis (4). Clinical examination findings in the retina of eyes that underwent ERG included mild peripheral tapetal mottling (4), slight retinal vascular attenuation (2), multifocal areas of altered tapetal reflectivity (2), peripapillary conus (1), mottling or pigment clumping in nontapetal fundus (2), focal choroidal hypoplasia (1), focal area of tapetal hyper-reflectivity (1). The fundus of two dogs contained a reduced amount of RPE/choroidal pigmentation (subalbinotic). The retina of one eye could not be clearly visualized due to cataract.

### 3.4. Electroretinography comparison with visual function questionnaire scores

Electroretinography were performed in 79 eyes from 79 dogs (35 right eyes and 44 left eyes). Spearman correlation coefficients and *p* values comparing visual function questionnaire responses with ERG b-wave amplitudes are presented in Table 2. Supplementary Figure S1 contains graphs of a subset of ERGs compared with visual function questionnaire responses.

**Table 2:** Comparison of dogVLQ subsections and VIS with ERG b-wave amplitudes.

Adaptation state	Light stimulus strength	Dog VLQ score Bright light	Dog VLQ score Dim light	Dog VLQ score near Darkness	VIS
Light-adapted	1 cds/m^2^	0.38 (*p* = 0.0006)	0.25 (*p* = 0.03)	0.17 (*p* = 0.15)	−0.26 (*p* = 0.02)
	3 cds/m^2^	0.26 (*p* = 0.02)	0.12 (*p* = 0.28)	0.16 (*p* = 0.16)	−0.30 (*p* = 0.008)
	10 cds/m^2^	0.17 (*p* = 0.14)	0.09 (*p* = 0.46)	0.16 (*p* = 0.15)	−0.26 (*p* = 0.02)
Dark-adapted	0.003 cds/m^2^	0.02 (*p* = 0.87)	0.09 (*p* = 0.43)	0.30 (*p* = 0.008)	−0.22 (*p* = 0.06)
	0.01 cds/m^2^	0.06 (*p* = 0.58)	0.08 (*p* = 0.47)	0.21 (*p* = 0.06)	−0.14 (*p* = 0.22)
	0.03 cds/m^2^	−0.0002 (*p* = 0.99)	−0.004 (*p* = 0.97)	0.17 (*p* = 0.13)	−0.10 (*p* = 0.38)
	0.1 cds/m^2^	−0.01 (*p* = 0.92)	−0.01 (*p* = 0.93)	0.16 (*p* = 0.15)	−0.14 (*p* = 0.22)
	0.3 cds/m^2^	0.03 (*p* = 0.79)	0.009 (*p* = 0.94)	0.19 (*p* = 0.09)	−0.20 (*p* = 0.08)
	1 cds/m^2^	0.09 (*p* = 0.43)	0.14 (*p* = 0.21)	0.24 (*p* = 0.03)	−0.22 (*p* = 0.06)
	3 cds/m^2^	0.09 (*p* = 0.44)	0.09 (*p* = 0.42)	0.25 (*p* = 0.03)	−0.22 (*p* = 0.06)
	10 cds/m^2^	0.10 (*p =* 0.39)	0.1 (*p* = 0.40)	0.23 (*p* = 0.04)	−0.15 (*p* = 0.19)

Spearman rank correlation *r* values and *p* values for ERG validation are shown for each dogVLQ subsection.

Adjusted scores for the Bright and Dim dogVLQ subsections were significantly associated with light-adapted b-wave amplitudes (Bright for 1 and 3 cds/m^2^, Dim for 1 cds/m^2^), whereas adjusted scores for the near Dark subsection were not associated with any light-adapted b-wave amplitude. Adjusted scores for the near Dark dogVLQ subsection were associated with dark-adapted dim flash and bright flash b-wave amplitudes (0.003, 1, 3, and 10 cds/m^2^) whereas the Bright and Dim subsections were not associated with any dark-adapted ERG amplitude. Adjusted VIS scores were significantly associated with all light-adapted responses and no dark-adapted ERG responses. Correlation r values for the Bright dogVLQ subsection ranged from −0.0002 to 0.10 for dark-adapted ERG responses, whereas r values for the VIS ranged from −0.14 to −0.22 for dark-adapted ERG responses (Table 2).

While owner responses for their dog’s vision in transitions between different light settings (bright to dim light, dim to bright light) were captured and compared with age, validation could not be performed as there was no ERG equivalent that could be compared.

### 3.5. Visual function questionnaire association with age

**Figure 1:**
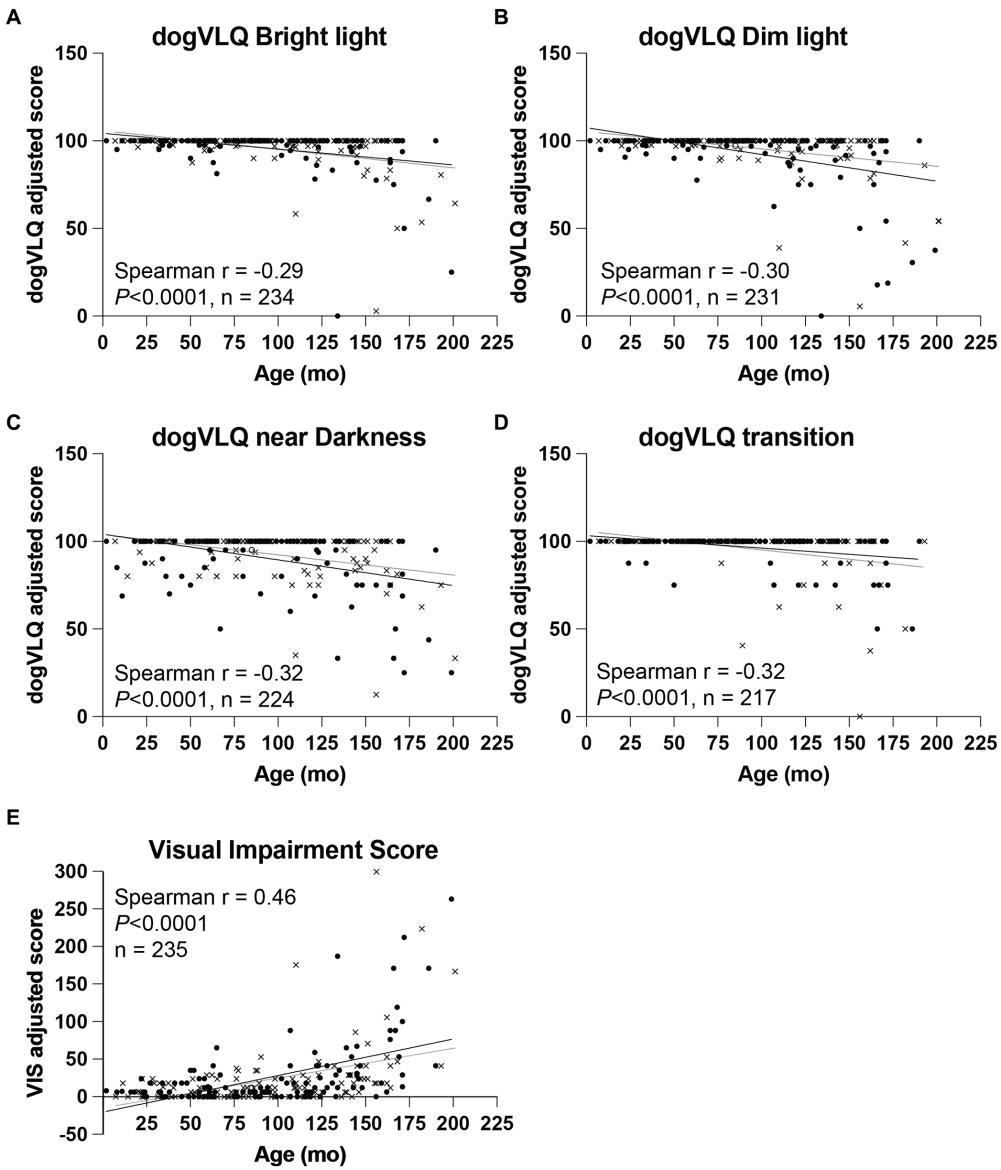
Dog variable lighting questionnaire (dogVLQ) subsection and VIS adjusted score association with dog age. Age was negatively associated with dogVLQ adjusted scores in bright light **(A)**, dim light **(B)**, near darkness **(C)**, and for transitions between lighting conditions **(D)**. Previously validated visual impairment score was also significantly associated with age **(E)**. Closed circles represent female dogs (*n* = 122), crosses represent male dogs (*n* = 112), open circle (*n* = 1) represents a dog which the owner did not disclose the sex. Black lines represent simple linear regression with age in females, gray lines in males; shown for illustration purposes only.

A significant negative association was found between age and dogVLQ responses for visually mediated behavior in bright light (*r* = −0.29, *p* < 0.0001, Figure 1A and Table 3), dim light (*r* = −0.30, *p* < 0.0001, Figure 1B and Table 3) and near darkness (*r* = −0.32, *p* < 0.0001, Figure 1C and Table 3). Age was also significantly associated with the individual dogVLQ transition scores (*r* = −0.30, *p* < 0.0001 bright light to dim light, data not shown and *r* = −0.33 *p* < 0.0001, dim to bright light, data not shown) and the overall transition score (*r* = −0.32, *p* < 0.0001, Figure 1D and Table 3). Older dogs also had worse vision as estimated by Visual Impairment Score (*r* = 0.46, *p* < 0.0001, Figure 1E). We performed age-associations with visual function questionnaire outcomes in each sex (male and female) separately, and there were no substantial differences (Table 3; male and female simple linear regression lines are shown in Figure 1 for illustration purposes). The non-normal distribution of the visual function questionnaire data precluded multivariable analysis on the basis of both sex and age. To account for the variation in anticipated lifespan between different breeds, a subset of dogs were assigned an estimated lifestage (from juvenile to geriatric). Lifestage was also associated with all dogVLQ subsections and the VIS (Table 3).

**Table 3:** Spearman rank correlation *r* values and (*p* values) are shown for each dogVLQ subsection and for VIS for comparisons with age in months, assigned dog lifestage and individually for male and female dogs.

	*n*	dogVLQ score bright light	dogVLQ score dim light	dogVLQ score near darkness	dogVLQ score transitions	VIS
Age (mo), all dogs	235	−0.29 (*p* < 0.0001)	−0.30 (*p* < 0.0001)	−0.32 (*p* < 0.0001)	−0.32 (*p* < 0.0001)	0.46 (*p <* 0.0001)
Age (mo), Male	112	−0.33 (*p* = 0.0008)	−0.27 (*p* = 0.01)	−0.32 (*p* = 0.002)	−0.36 (*p* = 0.0002)	0.45 (*p* < 0.0001)
Age (mo), Female	122	−0.25 (*p* = 0.005)	−0.32 (*p* = 0.0003)	−0.31 (*p* = 0.0006)	−0.28 (*p* = 0.003)	0.48 (*p* < 0.0001)
Life stage	147	−0.27 (*p* = 0.0007)	−0.30 (*p* = 0.0003)	−0.35 (*p <* 0.0001)	−0.27 (*p* = 0.001)	0.46 (*p* < 0.0001)

Because both the dogVLQ Bright and Dim subsections only correlated with light-adapted ERG, and represented identical questions with only lighting conditions differing, we hypothesized that owners responded similarly to both sets of questions, and that the scores for each of these subsections would be related. Spearman correlation analysis confirmed this hypothesis, with an r of 0.67, *p* < 0.0001. The Bright and Dark subsections (Spearman *r* = 0.46, *p* < 0.0001), Bright and Transition subsections (Spearman *r* = 0.48, *p* < 0.0001), and Dark and Transition subsections (Spearman *r* = 0.46, *p* < 0.0001) were less associated than the Bright and Dim subsections, indicating they captured different aspects of visual behavior.

Regression line slope and *Y* = 100 x-intercepts for the dogVLQ and *y* = 0 x-intercept for the VIS were calculated based on the simple linear regression formulae (Table 4). These x-intercepts represent the predicted estimated “age of onset” of decline in vision in the VIS or each dogVLQ lighting subsection. *Y* = 100 x-intercepts for the Bright and Dim dogVLQ subsections were similar (both 51 months) whereas the *Y* = 100 x-intercept for the near Dark subsection was younger (30 months). VIS estimated onset of decline was 40 months. We tested if the slope and x-intercepts of the dogVLQ Bright and near Dark regression lines with age were different. The slopes were not significantly different (*F* = 1.511. DFn = 1, DFd = 454, *p* = 0.2159), but the x-intercepts were significantly different (*F* = 11.28. DFn = 1, DFd = 4,555, *p* = 0.00). Similarly, we compared the slope and x-intercepts for the regression of age and bright light ERG b-wave amplitude (LA 3cds/m^2^) and dim light ERG b-wave amplitude (DA 0.003cds/m^2^). The slopes of these regressions were not significantly different (LA 3cds/m^2^ slope − 0.19, *p* < 0.0001; DA 0.003cds/m^2^ slope − 0.35, *p* = 0.002; *F* = 1.814. DFn = 1, DFd = 156, *p* = 0.18) but the x-intercepts were significantly different (LA 3cds/m^2^ Y-intercept 83.4 μV, X-intercept 436 months, DA 0.003cds/m^2^ Y-intercept 119.4 μV, X-intercept 342 months; *F* = 16.16. DFn = 1, DFd = 156. *p* < 0.0001). While association between age and ERG was not a primary endpoint of this study, all ERG b-wave amplitudes were associated with age (Spearman r values ranged from −0.29 to −0.58, *p* values ranged from 0.03 to <0.0001; data not shown).

**Table 4:** Simple linear regression slopes and x-intercepts for dogVLQ and VIS scores with dog age.

	dogVLQ score bright light	dogVLQ score dim light	dogVLQ score near darkness	VIS
Slope	−0.10	−0.13	−0.13	0. 45
X- (age) intercept at y (questionnaire score) = 100 for VLQ, *y* = 0 for VIS (95% confidence interval)	51 months* (21–69)	51 months (24–69)	30 months* (−5–51)	40 months (19–55)

* Indicates that *x* = 100 intercepts are significantly different from each other (*p* = 0.0009).

## 4. Discussion

This study describes the development and testing of the dog variable lighting questionnaire (dogVLQ), a proxy tool to quantify visually mediated behavior in dogs in a variety of light settings. We show that these measures are related to retinal function assessed using electroretinography (ERG). We found statistically significant associations between relevant ERG b-wave amplitudes and our dogVLQ scores. Both dogVLQ and previously published dog visual impairment score (VIS) measurements (22) provide evidence for age-associated visual dysfunction in dogs. Like humans, the predicted age of onset for decline of dog vision in very low lighting is earlier than predicted decline of vision in brighter lighting.

In the development of the dogVLQ, we made careful consideration to include relevant visually mediated behaviors and activities that companion dogs perform in different lighting conditions with their owners. Our content validation identified one question that did not meet content validity criteria, and additional feedback from experts identified additional visually mediated behaviors that were incorporated into the final questionnaire design. In dogVLQ question responses, owners were given options to explain if a dog did not perform a behavior at all or did not because of reasons unrelated to vision. This is an important consideration in older dogs that are more likely to have orthopedic, non-ophthalmic neurologic, or other health-related reasons (unrelated to vision) inhibiting performance of certain behaviors, for example navigating curbs or stairs. It is possible that other sensorineural inputs affect the owner’s responses to certain questions, for example olfactory detection of the treat might impact whether a dog looks at the owner’s hand when they are holding a treat and a dog’s hearing may affect their ability to make eye contact with an owner when called. We attempted to capture behaviors relevant to three different lighting conditions – bright lighting (equivalent to outside on a sunny day), dim lighting (equivalent to street lighting or dim indoor lighting), and near dark (outside at night with little to no lighting). We did not ask the owners to measure lighting conditions at home, but empirically, residential night street lighting [approximately 4 lux (36)] is at least 1,000 fold lower luminance than outdoor on a sunny day [approximately 10,000 lux (37)]. Street lighting is therefore likely to be sufficient to facilitate cone-mediated vision in dogs (38). We found that responses to the “bright” and “dim” lighting conditions were similar, and both subsections only associated with ERG responses in the light-adapted state, indicating that they were more associated with vision in brighter lighting conditions. Whether this reflects the similarities in questions (the set of questions for bright and dim were identical aside from the hypothetical lighting condition), or that the specific lighting environment described is not able to discriminate subtleties of vision differences is unclear. However, the fact that ERG amplitudes were similarly associated with responses in both subsections (light-adapted responses were associated, dark-adapted responses were not associated) indicates that similar visual behavioral responses were captured with each subsection. In addition, the responses to the bright and dim subsections were most strongly associated. In future studies, the dim subsection could be omitted due to overlap with the bright subsection responses. Because the other subsections were less substantially associated with each other, we recommend they are included, so the final questionnaire would include a score for Bright, Dark and Transitions subsections, comprising a total of 17 questions. The dogVLQ offers advantages over the VIS as it is capable of separating vision in bright light from vision in very low lighting, whereas the VIS more closely represents vision in bright light. This may be pertinent for early detection of hereditary retinal disorders that commonly affect either the rod, or cone photoreceptors initially, before proceeding in later stages to more generalized photoreceptor disease (23).

Both human and dog retinae are rod dominated, and contain a focal area of cone concentration, which is more pronounced in humans than in dogs (39–41). In aging humans, clinically significant decline in dim-light vision onsets earlier than bright-light vision is compromised (9). Using cross-sectional data analysis of slopes and x-intercepts of the association between age and ERG and dogVLQ subsections, we predict that dogs mirror humans in this regard. However, the exact cutoff for clinically significant visual deficits is not yet understood in dogs, and longitudinal studies are necessary to confirm our predicted age of onset of bright light and dim light visual decline in dogs. This estimated age of onset requires clinical validation with objective tools, and the clinical significance of mild dysfunctions identified using the questionnaire will need to be clarified if the questionnaire outcomes can be used in clinical decision making in veterinary medicine. Development of clinical vision tests for dogs that assess visual behavior in different luminance settings in a standardized manner will be an important future direction. These clinical tests would help to confirm that low luminance vision deficit onsets earlier than bright light vision deficit, and complement the emerging low luminance visual acuity (LLVA) tests developed for humans (10). A deficit identified using LLVA testing in humans typically onsets earlier in age-related retinal diseases than standard visual acuity testing, and therefore may be a more sensitive clinical outcome measure to study age-related visual sensory loss and efficacy of treatment. Clinical testing of vision in dogs is more challenging than in humans due to the lack of verbal feedback. However, a maze test (42), and a tunnel choice test (43) have been evaluated in a number of dog models of human hereditary eye diseases, and although time consuming to perform, could be utilized to objectively assess age-dependent changes in dog vision in different lighting settings.

Vision is a highly relevant sense for dogs (38). A recent study concluded that dogs rely on vision for object recognition and switch to other sensory modalities only when searching in the dark (44). Our research suggests that older dogs have difficulty using vision to navigate their environments when lighting is very limited. Because it is possible that olfaction (45) and hearing (46) are also impaired in older dogs, very low lighting conditions may be particularly difficult for older dogs to navigate in unfamiliar circumstances due to multisensory deficits limiting the ability of a dog to “switch” from one sense to another. Multisensory deficits contribute to an increase in abnormal behavior traits in dogs (47), namely increased tendency toward obsessive compulsive behaviors and reduced communication with owners. Dogs with multisensory deficits were also reported to participate less frequently in dog sports and activities (47). This suggests that multisensory deficits in dogs impact quality of life for both dog and owner. Multisensory deficits in older humans places them at significantly increased risk for dementia; impairment of both hearing and vision increases the risk of cognitive impairment by 8-fold (48), more than what is expected from the sum of each individual factor. Further research is necessary to determine if sensory deficits in older dogs are associated with worse cognitive function. Many clinical cognitive tests in dogs require integrated sensory/cognitive/motor activity, for example in the delayed nonmatching-to-position task (49), where the dog must view the location of a reward, remember its location after a delay, and move toward the anticipated location. The contribution of sensory dysfunction to cognitive test outcomes will be important to dissect as treatment modalities are developed and standardized outcome measures are utilized for clinical trials for sensory and cognitive dysfunction in both dogs and humans. Companion dogs could be important sentinels of aspects of the human lifestyle or home environment (shared with dogs) that contribute to accelerated aging. Use of outbred dogs in their natural home environment improves the chance of translation and reproducibility by considering aspects of the newly developed “STRANGE” animal behavior framework, whereby careful selection of diverse animal subjects for research studies improves reproducibility of outcomes in future studies (50).

We acknowledge some potential limitations of this work. Firstly, the dogVLQ instrument is a proxy subjective measure. However, proxy subjective instruments for assessment of dog cognition (51) and hearing (52) have been previously validated against clinical outcome measures and are established clinical research tools in veterinary medicine. Because the questionnaire is subjective, the human owner’s vision may also impact responses – i.e., if the owner has more difficulty seeing in specific lighting conditions, this may impact their perception of their dog’s vision. There is also likely variance in how “closely” different dog owners observe their dogs – some dogs may not go on regular walks with their owners, for example. We also acknowledge that we did not recruit severely visually impaired dogs for this study and the variation in responses was relatively small. However, our goal was to detect subtle vision impairment, as more significant impairment is more readily observed by dog owners, prompting them to seek veterinary advice. We also acknowledge that electroretinography is only measuring retinal function whereas the questionnaire responses relate to behaviors that integrate sensory input, cognition and motor function, all of which can be impacted by aging and disease. This is reflected in the moderate association between the visual function questionnaire responses and electroretinogram b-wave amplitudes, indicating that other factors influence perceived dog visually mediated behavior other than vision itself. However, similarly, the association between the visual function questionnaires and age (and ERG amplitudes and age) were also moderate, suggesting that age one of multiple factors influencing vision and visually-mediated behavior in dogs. Whilst we acknowledge potential limitations, our proxy dog owner visual assessment tool is very feasible, low cost, accessible, and may provide veterinarians with important complementary information on visual quality of life in their veterinary patients. This instrument captures complex integrated visually-mediated behaviors observable to the dog owner, and therefore could also be useful in assessing the potential therapeutic benefits of interventions for diseases that affect vision in dogs, particularly in clinical trials using client-owned dogs.

## Data availability statement

The raw data supporting the conclusions of this article will be made available by the authors, without undue reservation.

## Ethics statement

The animal studies were approved by University of Wisconsin-Madison Institutional Animal Care and Use Committee. The studies were conducted in accordance with the local legislation and institutional requirements. Written informed consent was obtained from the owners for the participation of their animals in this study.

## Author contributions

FM and NM secured the funding and conceived the study. CR and FM wrote the initial draft of this manuscript. FM and LR developed, pilot-tested, and revised the instrument. CR, MS, and FM performed the clinical procedures. MS, CR, and HL performed *post hoc* data generation (ERG analysis and data management). ZL performed statistical analysis of the study data. All authors contributed to the article and approved the submitted version.

## Funding

This work was funded by K08EY028628 to FM, Morris Animal Foundation Mark L. Morris Jr. Investigator Award D23CA-510 to FM, donor funds to the Department of Ophthalmology and Visual Sciences at the University of Wisconsin-Madison and supported in part by an Unrestricted Grant from Research to Prevent Blindness, Inc. to the UW-Madison Department of Ophthalmology and Visual Sciences. Statistical support was funded by a National Center for Advancing Translational Sciences, Grant/Award Number: UL1TR002373.

## Conflict of interest

The authors declare that the research was conducted in the absence of any commercial or financial relationships that could be construed as a potential conflict of interest.

## Publisher’s note

All claims expressed in this article are solely those of the authors and do not necessarily represent those of their affiliated organizations, or those of the publisher, the editors and the reviewers. Any product that may be evaluated in this article, or claim that may be made by its manufacturer, is not guaranteed or endorsed by the publisher.

## Footnotes

^1^ researchrandomizer.com
