# Supplementary material for: Subjective vision assessment in companion dogs using dogVLQ demonstrates age-associated visual dysfunction
Source: Front Vet Sci. 2023 Aug 17;10:1244518. doi: 10.3389/fvets.2023.1244518 (PMC10469761; doi:10.3389/fvets.2023.1244518)
Supplement: Supplementary file 1 [file Data_Sheet_1.docx]

**Low luminance vision questionnaire**

These questions relate to your opinion on how your dog behaves in specific lighting conditions, as a direct result of their vision. In each section, we provide a short summary of the lighting conditions we are asking about. Please read each question carefully – in order for us to understand how different lighting conditions affect your dog, your answers must be as accurate as possible. Please take as much time as you need to answer each question (you may wish to try out some scenarios with your dog if they are unfamiliar). Please choose the response that describes your situation at the present time. There are 4 subsections.

Please remember that your dog will often “learn” it’s home environment very well. It is best to imagine or describe how your dog performs in less familiar environments – for example when out for a walk, at the dog park or in a friend or neighbor’s home or yard.

You will rate your dog’s difficulty with certain activities/behaviors from easiest to hardest. These options will be listed first (up to 5 choices).

If your dog does not do the activity or has trouble with it because of conditions NOT related to vision (for example orthopedic or arthritis issues and has trouble with stairs or steps because of that), please select: “My dog cannot do (or has difficulty with) this activity for reasons other than their vision”

If you have not seen the activity performed by your dog or your dog just doesn’t do that activity (for example giving a high five or a paw), please select: “I do not do this activity with my dog or my dog does not do this activity”

________________________________________________________________________________________

**Bright lighting conditions**

These questions ask about your dog's behavior in bright lighting conditions. An example of bright lighting includes outside on a sunny day.

**1. [BLVIS]** In general, does your dog have difficulty seeing in bright sunlight? Select ONE.

No difficulty at all

A little difficulty

Some difficulty

A lot of difficulty

My dog cannot do this activity due to their vision

My dog cannot do (or has difficulty with) this activity for reasons other than their vision

I do not do this activity with my dog or my dog does not do this activity

**2. [BLEYECONTACT]**In bright sunlight, when your face is well lit, does your dog have difficulty making eye contact (or looking at your face) if you try to get their attention? Select ONE.

No difficulty at all

A little difficulty

Some difficulty

A lot of difficulty

My dog cannot do this activity due to their vision

My dog cannot do (or has difficulty with) this activity for reasons other than their vision

I do not do this activity with my dog or my dog does not do this activity

**3. [BLBACKLIT1]**In bright sunlight when the sun is behind you (you are backlit and your face is shaded), does your dog have difficulty making eye contact (or looking at your face)? Select ONE.

No difficulty at all

A little difficulty

Some difficulty

A lot of difficulty

My dog cannot do this activity due to their vision

My dog cannot do (or has difficulty with) this activity for reasons other than their vision

I do not do this activity with my dog or my dog does not do this activity

**4. [BLTOYTRACK]**In bright sunlight, does your dog have difficulty tracking a toy when thrown? By tracking we mean following the toy with their eyes or head when the toy is thrown in the air or along the ground. Select ONE.

No difficulty at all

A little difficulty

Some difficulty

A lot of difficulty

My dog cannot do this activity due to their vision

My dog cannot do (or has difficulty with) this activity for reasons other than their vision

I do not do this activity with my dog or my dog does not do this activity

**5. [BLCATCH]**In bright sunlight, does your dog have difficulty catching an object from the air when thrown? This can be a toy or a treat. Select ONE.

No difficulty at all

A little difficulty

Some difficulty

A lot of difficulty

My dog cannot do this activity due to their vision

My dog cannot do (or has difficulty with) this activity for reasons other than their vision

I do not do this activity with my dog or my dog does not do this activity

**6. [BLTREAT]**In bright sunlight, does your dog look at your hand when you have a treat or toy in it? Select ONE.

Yes

No

I don’t know

I do not do this activity with my dog or my dog does not do this activity

**7. [BLPAW]**In bright sunlight, does your dog have difficulty accurately giving you a “high five” or a paw? By accurately we mean they hit the target of your hand correctly without missing. Select ONE.

No difficulty at all

A little difficulty

Some difficulty

A lot of difficulty

My dog cannot do this activity due to their vision

My dog cannot do (or has difficulty with) this activity for reasons other than their vision

I do not do this activity with my dog or my dog does not do this activity

**8. [BLDOWNSTAIRS]**In bright sunlight, when out for a walk (or in less familiar surroundings) does your dog have difficulty going DOWN curbs or stairs because of their vision? Difficulty might look like hesitance, being slow or deliberate, refusal, vocalization to get help, or misjudging height and stumbling/falling. Try to think of their actions when out for a walk in an unfamiliar environment, rather than at home. Remember that we are asking specifically about difficulty because of vision. Select ONE.

No difficulty at all

A little difficulty

Some difficulty

A lot of difficulty

My dog cannot do this activity due to their vision

My dog cannot do (or has difficulty with) this activity for reasons other than their vision

I do not do this activity with my dog or my dog does not do this activity

**9. [BLPERIPH]**In bright sunlight, does your dog have difficulty seeing moving objects in their  peripheral vision (objects coming at them from the side)? For example, a ball rolling from their side. Select ONE.

No difficulty at all

A little difficulty

Some difficulty

A lot of difficulty

My dog cannot do this activity due to their vision

My dog cannot do (or has difficulty with) this activity for reasons other than their vision

I do not do this activity with my dog or my dog does not do this activity

**10. [BLFAMILIAR]**In bright sunlight, does your dog have difficulty recognizing familiar objects if they are not in their usual place? For example, your dog gets startled by, or barks at a trash can or a plant stand that has been moved out of its usual place. Select ONE.

No difficulty at all

A little difficulty

Some difficulty

A lot of difficulty

My dog cannot do this activity due to their vision

My dog cannot do (or has difficulty with) this activity for reasons other than their vision

I do not do this activity with my dog or my dog does not do this activity

___________________________________________________________________________________________________________

**Dim lighting conditions:**

These questions ask about your dog's behavior in dim lighting conditions. Examples of this type of lighting include outside at dusk or dawn or inside in a poorly lit room (no overhead lights on).

**12. [DLVIS]** In general, does your dog have difficulty seeing in dim lighting? Select ONE.

No difficulty at all

A little difficulty

Some difficulty

A lot of difficulty

My dog cannot do this activity due to their vision

My dog cannot do (or has difficulty with) this activity for reasons other than their vision

I do not do this activity with my dog or my dog does not do this activity

**13. [DLEYECONTACT]**In dim lighting, does your dog have difficulty making eye contact (or looking at your face) if you try to get their attention? Select ONE.

No difficulty at all

A little difficulty

Some difficulty

A lot of difficulty

My dog cannot do this activity due to their vision

My dog cannot do (or has difficulty with) this activity for reasons other than their vision

I do not do this activity with my dog or my dog does not do this activity

**14. [DLBACKLIT2]** When you are in dim lighting (for example a dark hallway or room) and sunlight or a bright light is behind you (you are backlit and your face is shaded), does your dog have difficulty making eye contact (or looking at your face)? Select ONE.

No difficulty at all

A little difficulty

Some difficulty

A lot of difficulty

My dog cannot do this activity due to their vision

My dog cannot do (or has difficulty with) this activity for reasons other than their vision

I do not do this activity with my dog or my dog does not do this activity

**15. [DLTOYTRACK]**In dim lighting, does your dog have difficulty tracking a toy when thrown? By tracking we mean following the toy with their eyes or head when the toy is thrown in the air or along the ground. Select ONE.

No difficulty at all

A little difficulty

Some difficulty

A lot of difficulty

My dog cannot do this activity due to their vision

My dog cannot do (or has difficulty with) this activity for reasons other than their vision

I do not do this activity with my dog or my dog does not do this activity

**16. [DLCATCH]**In dim lighting, does your dog have difficulty catching an object from the air when thrown? This can be a toy or a treat. Select ONE.

No difficulty at all

A little difficulty

Some difficulty

A lot of difficulty

My dog cannot do this activity due to their vision

My dog cannot do (or has difficulty with) this activity for reasons other than their vision

I do not do this activity with my dog or my dog does not do this activity

**17. [DLTREAT]**In dim lighting, does your dog look at your hand when you have a treat or toy in it? Select ONE.

Yes

No

I don’t know

I do not do this activity with my dog or my dog does not do this activity

**18. [DLPAW]**In dim lighting, does your dog have difficulty accurately giving you a “high five” or a paw? By accurately we mean they hit the target of your hand correctly. Select ONE.

No difficulty at all

A little difficulty

Some difficulty

A lot of difficulty

My dog cannot do this activity due to their vision

My dog cannot do (or has difficulty with) this activity for reasons other than their vision

I do not do this activity with my dog or my dog does not do this activity

**19. [DLDOWNSTAIRS]**In dim lighting, when out for a walk (or in less familiar surroundings) does your dog have difficulty with going down curbs or stairs because of their vision? Difficulty might look like hesitance, being slow or deliberate, refusal, vocalization to get help, or misjudging height and stumbling/falling.  Try to think of their actions when out for a walk in an unfamiliar environment, rather than at home. Remember that we are asking specifically about difficulty because of vision. Select ONE.

No difficulty at all

A little difficulty

Some difficulty

A lot of difficulty

My dog cannot do this activity due to their vision

My dog cannot do (or has difficulty with) this activity for reasons other than their vision

I do not do this activity with my dog or my dog does not do this activity

**20. [DLPERIPH]**In dim lighting, does your dog have difficulty seeing moving objects in their peripheral vision (objects coming at them from the side)? For example, a ball rolling from their side. Select ONE.

No difficulty at all

A little difficulty

Some difficulty

A lot of difficulty

My dog cannot do this activity due to their vision

My dog cannot do (or has difficulty with) this activity for reasons other than their vision

I do not do this activity with my dog or my dog does not do this activity

**21. [DLFAMILIAR]**In dim lighting, does your dog have difficulty recognizing familiar objects if they are not in their usual place? For example, your dog gets startled by, or barks at a trash can or a plant stand that has been moved out of its usual place. Select ONE.

No difficulty at all

A little difficulty

Some difficulty

A lot of difficulty

My dog cannot do this activity due to their vision

My dog cannot do (or has difficulty with) this activity for reasons other than their vision

I do not do this activity with my dog or my dog does not do this activity

**Dark conditions**

These questions ask about your dog's behavior in very low lighting conditions or in almost darkness. Examples of this type of lighting includes at night outdoors when out for a walk or in the yard at night with little to no artificial lighting.

**22. [DARKVIS]** Does your dog have difficulty seeing in darkness (at night with little to no lighting)? Select ONE.

No difficulty at all

A little difficulty

Some difficulty

A lot of difficulty

My dog cannot do this activity due to their vision

My dog cannot do (or has difficulty with) this activity for reasons other than their vision

I do not do this activity with my dog or my dog does not do this activity

**23. [DARKDOWNSTAIRS]**In darkness (at night with little to no lighting), when out for a walk (or in unfamiliar surroundings) does your dog have difficulty with going down curbs or stairs because of their vision? Try to think of their actions when out for a walk in an unfamiliar environment, rather than at home. Select ONE.

No difficulty at all

A little difficulty

Some difficulty

A lot of difficulty

My dog cannot do this activity due to their vision

My dog cannot do (or has difficulty with) this activity for reasons other than their vision

I do not do this activity with my dog or my dog does not do this activity

**24. [DARKFLASHLIGHT]**In darkness (at night with little to no lighting), can your dog chase or follow a flashlight or headlamp around? Select ONE.

All of the time

Most of the time

Some of the time

A little of the time

None of the time

I do not do this activity with my dog or my dog does not do this activity

**25. [DARKDEPEND]**In darkness (at night with little to no lighting), do you have to help your dog more because of their vision? For example lifting them up or down stairs, or in/out of the car. Select ONE.

None of the time

A little of the time

Some of the time

Most or all of the time

I stopped doing this activity because my dog’s vision causes them to be too dependent on me

My dog cannot do (or has difficulty with) this activity for reasons other than their vision

I do not do this activity with my dog or my dog does not do this activity

**26. [DARKOUT]**In darkness (at night with little to no lighting), do you limit taking your dog out because of their vision?  Select ONE.

None of the time

A little of the time

Some of the time

Most of the time

All of the time

I do not do this activity with my dog or my dog does not do this activity

**Transitions between lighting conditions**

These questions relate to activities when your dog has to move between different lighting conditions – for example moving between outdoors (bright sunshine) and indoors (dark indoor room). Please read the question carefully to make sure you understand the transition correctly.

**27. [BLTODL]**When transitioning from bright lighting to dim lighting, does your dog have difficulty navigating (for example they stumble, trip or hesitate on steps or doorways)?  Example of this type of lighting transition is coming indoors from outdoors when there is bright sunlight outdoors. Select ONE.

No difficulty at all

A little difficulty

Some difficulty

A lot of difficulty

My dog cannot do this activity due to their vision

My dog cannot do (or has difficulty with) this activity for reasons other than their vision

I do not do this activity with my dog or my dog does not do this activity

**28. [DLTOBL]**When transitioning from dim lighting to bright lighting, does your dog have difficulty navigating (for example they stumble, trip or hesitate on steps or doorways)? Example of this type of lighting transition is going outdoors from indoors when there is bright sunlight outdoors. Select ONE.

No difficulty at all

A little difficulty

Some difficulty

A lot of difficulty

My dog cannot do this activity due to their vision

My dog cannot do (or has difficulty with) this activity for reasons other than their vision

I do not do this activity with my dog or my dog does not do this activity

Questions excluded from final questionnaire after content validation exercise.

When taking your dog for a walk, if your dog has to navigate outside porch or front steps at both the beginning and end of a walk, which of the following statements applies?

Question removed because it failed content validation

1. Both beginning and end of walk
2. Neither beginning or end of walk
3. better at beginning of walk
4. better at end of walk,
5. My dog refuses both,
6. does not apply

At night (very low lighting conditions), does your dog have difficulty making eye contact with you if you make a noise or call their name to get their attention?

Question removed due to expert feedback that dogs will unlikely perform this behavior in this lighting condition

1.     No difficulty at all
2.     A little difficulty
3.     Some difficulty
4.     A lot of difficulty
5.     My dog cannot do this activity in these conditions
6.     I stopped doing this activity in these conditions because of my dog’s vision
7.     I stopped doing this activity in these conditions because of other reasons  
8.     I have never done this activity with my dog in these conditions

At night (very low lighting conditions), does your dog have difficulty tracking an object when thrown (for example a toy or treat)?

Question removed due to expert feedback that dogs will unlikely perform this behavior in this lighting condition

1.     No difficulty at all
2.     A little difficulty
3.     Some difficulty
4.     A lot of difficulty
5.     My dog cannot do this activity in these conditions
6.     I stopped doing this activity in these conditions because of my dog’s vision
7.     I stopped doing this activity in these conditions because of other reasons  
8.     I have never done this activity with my dog in these conditions

At night (very low lighting conditions), does your dog have difficulty seeing moving objects in their peripheral vision (objects coming at them from the side)?

Question removed due to expert feedback that dogs will unlikely perform this behavior in this lighting condition

1.     No difficulty at all
2.     A little difficulty
3.     Some difficulty
4.     A lot of difficulty
5.     My dog cannot do this activity in these conditions
6.     I stopped doing this activity in these conditions because of my dog’s vision
7.     I stopped doing this activity in these conditions because of other reasons  
8.     I have never done this activity with my dog in these conditions

At night (very low lighting conditions), does your dog have difficulty recognizing familiar objects (for example a trash can or plant stand in its usual position)?

Question removed due to expert feedback that dogs will unlikely perform this behavior in this lighting condition

1.     No difficulty at all
2.     A little difficulty
3.     Some difficulty
4.     A lot of difficulty
5.     My dog cannot do this activity in these conditions
6.     I stopped doing this activity in these conditions because of my dog’s vision
7.     I stopped doing this activity in these conditions because of other reasons  
8.     I have never done this activity with my dog in these conditions
