# Supplementary material for: Subjective vision assessment in companion dogs using dogVLQ demonstrates age-associated visual dysfunction
Source: Front Vet Sci. 2023 Aug 17;10:1244518. doi: 10.3389/fvets.2023.1244518 (PMC10469761; doi:10.3389/fvets.2023.1244518)
Supplement: Supplementary file 2 [file Image_1.pdf]

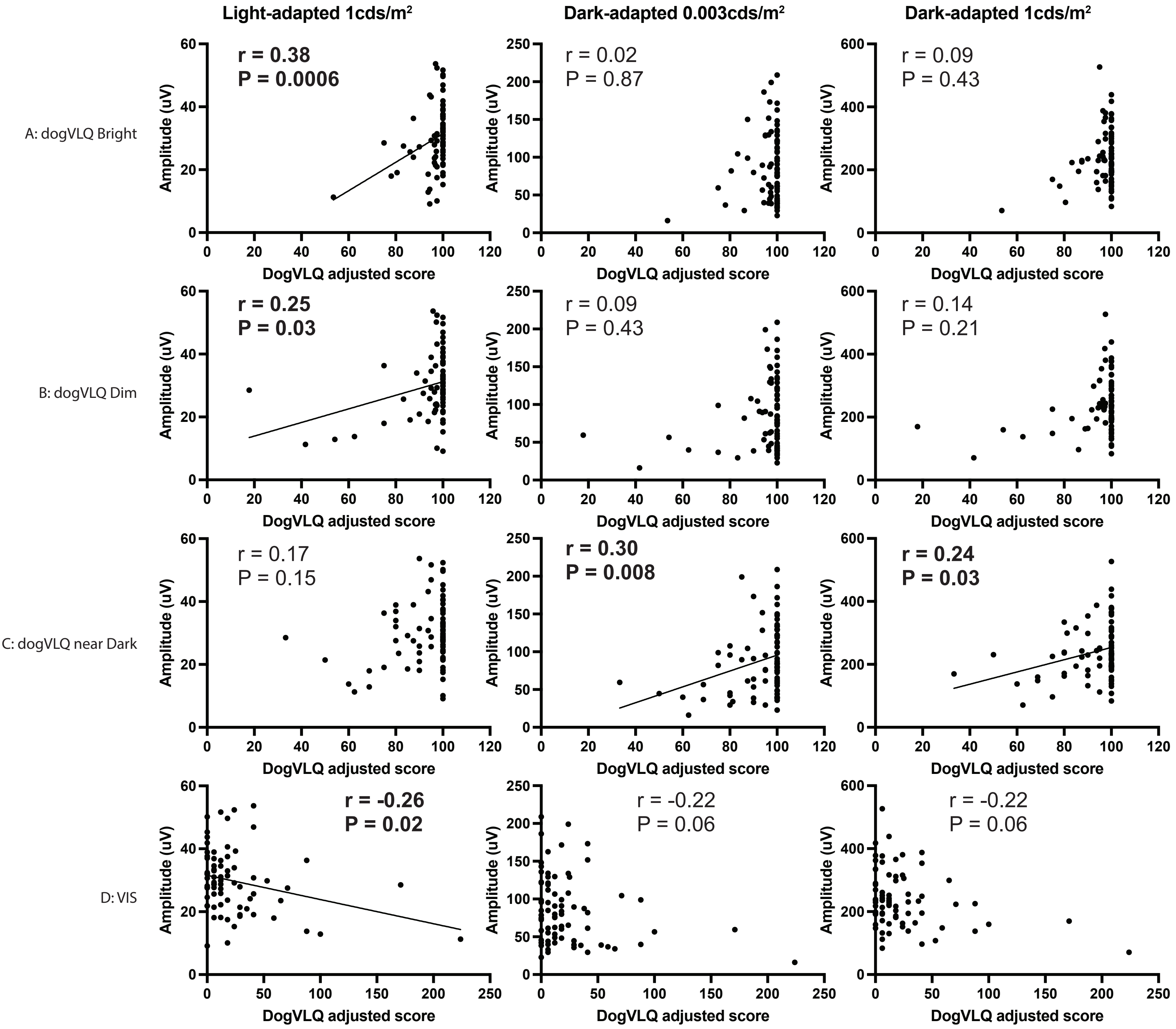

Supplementary figure 1. X-Y scatter plots shown for the association between visual function questionnaires (x axis) and electroretinogram b-wave amplitudes (y axis) for each of the questionnaire subsections of the dogVLQ and the VIS. Spearman rank correlation p values and r values are shown, bolded if significant. Simple linear regression lines are shown for illustration purposes for significantly associated parameters.
